# Radiotherapy medical physics in the Philippines: A contemporary overview

**DOI:** 10.1002/acm2.70129

**Published:** 2025-05-31

**Authors:** John Paul C. Cabahug, Ramon Carlo Cruzpero, Luis E. Fong de los Santos, Eric C. Ford, Afua A. Yorke

**Affiliations:** ^1^ Department of Medical Physics University of Wisconsin−Madison Madison Wisconsin USA; ^2^ Health Physics Research Section Atomic Research Division (ARD‐HPRS) DOST‐PNRI Quezon City Republic of the Philippines; ^3^ Department of Radiology University of the Philippines–Philippine General Hospital Ermita, Manila Philippines; ^4^ Department of Radiation Oncology Mayo Clinic Rochester Minnesota USA; ^5^ Department of Radiation Oncology Fred Hutch Cancer Center University of Washington School of Medicine Seattle Washington USA

**Keywords:** dosimetric tool availability, quality assurance, radiotherapy equipment, radiotherapy safety practices, treatment interruptions

## Abstract

**Purpose:**

With cancer ranking as the third leading cause of death in the Philippines and a disparity in healthcare resources across regions, this research aimed to assess the state of radiotherapy medical physics in the country.

**Methodology:**

The study utilized a comprehensive online survey with 94 structured questions answered by 19 clinics.

**Results:**

Most of the participants were within 1–3 years of training (41%), with a slight majority working in private hospitals (55%). linear accelerators (LINACs) were universally used with one Co‐60 unit available, and High Dose Rate (HDR) brachytherapy was common. Intensity‐Modulated Radiotherapy (IMRT) and 3D‐Conformal Radiotherapy (3D‐CRT) are practiced in all 19 clinics, with advanced techniques like Stereotactic Body Radiotherapy (SBRT), Stereotactic Radiosurgery (SRS), and Intraoperative Radiotherapy (IORT) limited to NCR, while modalities such as Volumetric Modulated Arc Therapy (VMAT) (21%) and 2D RT (68%) are more widely practiced. Imaging modalities included the wide adoption of Computed Tomography (CT), though only 64% of respondents had dedicated CT simulators in their clinics. Gynecologic and breast cancers were frequently treated, while bone marrow transplants (total body irradiation) were rare. For quality assurance (QA) devices, Solid Water Phantoms and Scanning Water Tanks (86%) were the most common devices for dosimetry and measurement. 82% reported performing patient‐specific QA (PSQA), with EPID dosimetry being the most common (55%) PSQA device used. Quality management practices varied between Qualified Medical Physicists and Medical Physics Trainees, with most Qualified Medical Physicists performing routine checks. Treatment interruptions were mainly due to staffing and machine downtime, rather than power outages or natural disasters. Most clinics had their own systems (86%) to document safety incidents, but only a few reported incidents (32%) to the IAEA SAFRON program. Lastly, participants expressed a willingness to collaborate in research despite limited time.

**Conclusion:**

This study provides an understanding of the current landscape of radiation therapy physics in the Philippines, highlighting the need to address workforce disparities, ensure equitable cancer treatment access, optimize dosimetric tools and QA devices, and prioritize resource allocation and research collaboration to advance radiation oncology practices.

## 1 INTRODUCTION

Cancer is a global health challenge, responsible for almost 10 million deaths in 2020, making it a leading cause of mortality worldwide.^1^ In the Philippines, it ranks as the third most common cause of death, claiming nearly 92 600 lives in 2020.^2^ Six out of every 10 (6 out of 10) Filipinos do not receive essential medical attention when facing this disease.^3^ Adding to the complexity, the Philippines, a lower‐middle‐income country in Southeast Asia, grapples with the unequal distribution of healthcare resources, especially among its 7600‐plus islands.^4^

The history of radiation therapy in the Philippines dates back to 1910 when the first treatment was given at the Philippine General Hospital. The equipment at the time included x‐ray tubes, Coolidge tubes, and a radium emanation room, just 14 years after Roentgen's discovery of x‐rays. In 1955, Manila Doctors Hospital, a private institution, became the first in the country to obtain an operational Co‐60 teletherapy unit. In 1962, two teletherapy units (a Co‐60 and a Cs‐137 unit) were installed at the Philippine General Hospital. A major milestone was achieved in 1983 with the installation of the first linear accelerator (LINAC) at the Lung Center of the Philippines.^4^, ^5^ However, despite a population of nearly 110 million, the Philippines faces a scarcity of medical specialists, with uneven distribution across regions, there are a total of 348 medical oncologists, 164 surgical oncologists, 99 radiation oncologists, 142 gynecologic oncologists, and 35 hospice and palliative medicine (HPM) specialists. Breaking this down per 100,000 Filipinos, it equates to approximately 0.32 medical oncologists, 0.15 surgical oncologists, 0.09 radiation oncologists, 0.13 gynecologic oncologists, and 0.03 HPM specialists.^3^ Breast cancer has emerged as the most common cancer type, contributing to a surge in cases that strains healthcare resources.^6^ Late‐stage cancer diagnoses in remote areas, limited access to advanced radiation therapy, and the financial burden of cancer care further compound the challenges^4^, ^7^

As of 2023, there are 51 radiotherapy centers in the Philippines, but access varies based on economic status.^7^ To address the pressing need for improved cancer care in resource‐constrained regions, some have worked on developing cost‐effective radiotherapy technologies for low‐ and middle‐income countries (LMICs),^8^, ^9^, ^10^ while others have focused on closing equipment gaps through donations.^11^, ^12^, ^13^ Ensuring the safety and quality of radiotherapy services, primarily overseen by medical physicists, is of utmost importance.^14^, ^15^, ^16^ The importance of establishing a comprehensive Quality Assurance (QA) program is highlighted in the report by the American Association of Physicists in Medicine (AAPM) Task Group 142. The report emphasizes the need for radiotherapy facilities to implement such a program tailored to their specific goals and resources.^17^ In addition to TG‐142, AAPM Task Group 198 provides a detailed implementation guide for TG‐142, focusing on QA procedures for medical accelerators.^18^ Medical Physics Practice Guideline (MPPG) 8a and 8b outline specific performance tests and updates for linear accelerators, ensuring safety and quality in clinical applications.^19^, ^20^

Many studies have investigated the challenges and needs of improving radiotherapy resources in Asia and LMICs. These studies emphasize two key points: the need for a strong team of medical physics professionals and the essential importance of making the best use of radiotherapy facilities.^15^, ^21^, ^22^, ^23^, ^24^, ^25^, ^26^, ^27^, ^28^ The objective of this study is to investigate and assess the current landscape of radiotherapy medical physics through the lens of QA and safety practices within healthcare facilities in the Philippines. This study employs a comprehensive survey methodology to investigate and address the current state of these practices, enabling the identification of challenges, gaps, and potential areas for improvement.

## 2 METHODOLOGY

### 2.1 Survey

This study utilized a survey‐based methodology to comprehensively investigate the landscape of medical physics QA and safety practices among the 19 radiotherapy centers in the Philippines. Out of the 51 radiotherapy centers in the country, nine (9) clinics from Luzon, and five (5) clinics each from Visayas and Mindanao responded to the survey. The questionnaire comprised a structured set of 94 questions, strategically designed to comprehensively understand the background and qualifications of the respondents, the availability of essential equipment, institutional practices, medical physics QA, quality management, safety practices, and research. The survey was designed by expert medical physicists with several years of experience. Ethical clearance for the survey was obtained from the Institutional Review Board, ensuring adherence to ethical standards throughout the research process. To facilitate data collection, the survey questionnaire was disseminated to multiple radiotherapy facilities via an online platform, specifically utilizing the Research Electronic Data Capture (REDCap) system. Prior to their participation, all respondents were provided with clear information regarding the study's objectives, the assurance of data confidentiality, and their unequivocal right to withdraw from the study at any point. Respondents were asked to rate the frequency of various aspects, including disease types treated, secondary monitor units (MU) calculation checks, and operational challenges affecting clinical practice. These ratings were collected using a 5‐point Likert system (Never = 1, Rarely = 2, Sometimes = 3, Often = 4, Always = 5). Quantitative data from the survey responses were analyzed using descriptive statistics. GraphPad software was used generate the figures.

## 3 RESULTS

### 3.1 Medical physics training and employment

Among the 22 participants in the study, the distribution of experience levels was as follows: three (3) medical physicists were currently in training, nine (9) were within 1–3 years post training, five (5) were within 4–7 years post training, four (4) were within 8–15 years post training, and one (1) had over 15 years of experience. Out of the 22 medical physicists who worked in radiotherapy, two (2) of them also worked in nuclear medicine or diagnostic imaging. A majority (12) of the physicists were employed in private hospitals while the remaining (10) had appointments in public hospitals.

### 3.2 Treatment modalities (Linear accelerator, Co‐60 teletherapy, and brachytherapy)

**FIGURE 1:**
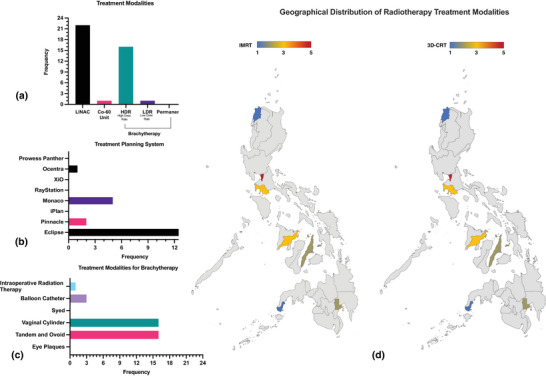
(a–d) Frequency distribution of the utilization of LINAC, Co‐60 teletherapy, and brachytherapy: HDR, LDR, and Permanent (a), TPS for radiotherapy (b), brachytherapy practices (c), and geographical distribution of radiotherapy practices (IMRT and 3D‐CRT) among 19 Clinics (d). 3D‐CRT, 3D‐Conformal Radiotherapy; HDR, high dose rate; IMRT, intensity‐modulated radiotherapy; LDR, low dose rate; LINAC, linear accelerators; TPS, treatment planning system.

Figure 1a shows the availability of teletherapy (LINACs and Co‐60 Units) and brachytherapy units (HDR—High Dose Rate and LDR—Low Dose Rate) in 19 clinics. Figure 1b shows the most used treatment planning system and Figure 1c illustrates the most common brachytherapy modality. Eclipse (68%) is the most employed TPS among clinics. Tandem and ovoid, along with vaginal cylinders, were commonly employed while eye plaques and Syed catheters were not common. The geographical distribution of Intensity‐Modulated Radiotherapy (IMRT) and 3D‐Conformal Radiotherapy (3D‐CRT) practices are shown in Figure 1d. IMRT is available in all 19 clinics, with NCR alone accounting for 26 % (5 clinics). 3D‐CRT is practiced in all 19 clinics across regions, with NCR contributing 26 % (5 clinics). The Visayas regions, particularly VI and VII, account for 26 % (5 clinics) of IMRT and 3D‐CRT practices. Mindanao regions (IX‐XII) presented 26% (5 clinics) of both IMRT and 3D‐CRT practices. Volumetric Modulated Arc Therapy (VMAT) is available in 21% (4 clinics) of the total facilities surveyed. It is practiced across four regions: NCR (Luzon), Region I (Luzon), Region VI (Visayas), and Region XII (Mindanao), with each region contributing 5 % (1 clinic). Traditional 2D Radiotherapy (2D RT) is utilized in 68 % (13 clinics) of facilities, with NCR providing 21% (4 clinics) of this modality. Advanced techniques like Stereotactic Body Radiotherapy (SBRT) and Stereotactic Radiosurgery (SRS) presented 11% (2 clinics) of facilities, both exclusively in NCR. Intraoperative Radiotherapy (IORT) and Total Body Irradiation (TBI) is limited to NCR (Luzon), representing 5% (1 clinic) of the total facilities. Total Skin Electron Therapy (TSE) and Proton Therapy are still absent in all surveyed regions.

### 3.3 Imaging modalities

**FIGURE 2:**
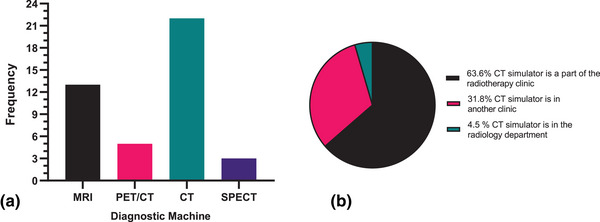
(a,b) Frequency distribution of the utilization of imaging modalities (MRI, PET/CT, CT, SPECT) (a) and location of CT simulator in radiation oncology clinics (b). MRI, Magnetic Resonance Imaging; PET/CT, Positron Emission Tomography/Computed Tomography; SPECT, Single Photon Emission Computed Tomography.

Figure 2 shows the utilization of various imaging modalities for staging, diagnosing, and scanning patients in the clinics. These modalities include, Magnetic Resonance Imaging (MRI), Positron Emission Tomography/Computed Tomography (PET/CT), CT, and Single Photon Emission Computed Tomography. CT (SPECT) was reported to be used by all 22 respondents (100%). Whiles Figure 2b shows the percentage of clinics that have dedicated in‐house CT simulators. It was reported that 63% had the CT simulator as being part of the radiotherapy clinic while 32% indicated that it was situated in another clinic and 5% indicated it was located in the radiology department.

### 3.4 Quality management and safety practices

**FIGURE 3:**
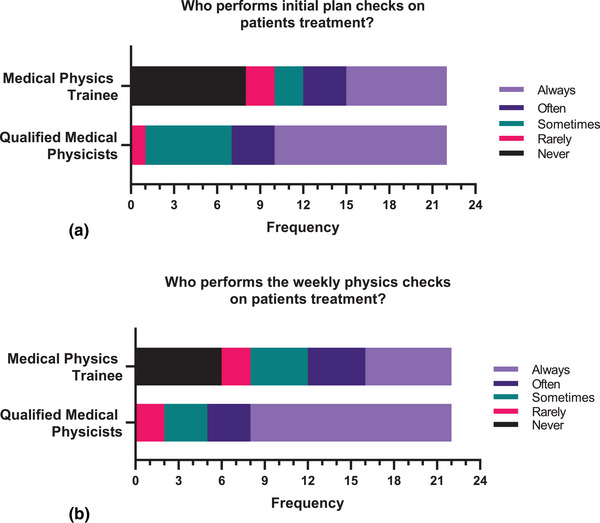
(a,b) Frequency distribution of the individuals responsible (Medical Physics Trainee or Qualified Medical Physicists) for the initial plan checks (a) and weekly physics checks (b) on patients' treatments undergoing radiotherapy using a 5‐point Likert scale (1 = Never, 2 = Rarely, 3 = Sometimes, 4 = Often, 5 = Always).

Figure 3a shows the data concerning initial plan checks for external beam patients in radiation therapy, focusing on Qualified Medical Physicists and Medical Physics Trainees. Among Qualified Medical Physicists, the majority (55%) reported performing QA checks “Always,” with a smaller percentage (14%) indicating “Often.” Additionally, 27% mentioned performing QA checks “Sometimes,” while only 5% reported doing so “Rarely.” Notably, none of the Qualified Medical Physicists reported performing QA checks “Never.” In contrast, Medical Physics Trainees showed a different distribution, with a significant portion (36%) reporting that they perform QA checks “Never.” Meanwhile, 32% of Medical Physics Trainees stated they perform QA checks “Always,” while 14% reported “Often.” Moreover, a combined 9.1% of Medical Physics Trainees indicated performing QA checks “Sometimes” and “Rarely.”

Figure 3b pertains to the individuals responsible for performing weekly physics checks on patients undergoing treatment in radiation therapy. For Qualified Medical Physicists, 14 out of 22 (64%), reported that weekly physics checks on patients' treatment are “Always”. Three respondents (14%) reported that these physics checks are conducted “Sometimes” and “Often”. Two respondents (9%) reported that these physics checks are conducted “Rarely.” None of the respondents indicated that weekly physics checks on patients' treatment are “Never”. For Medical Physics Trainees, six respondents (27%) indicated that weekly physics checks on patients' treatment are “Always” and “Never”. Four respondents (18%) reported that these physics checks are conducted “Often” and “Sometimes.” Two respondents (9%) indicated that these physics checks are conducted “Rarely”.

In determining the various factors that contribute to treatment interruptions in a radiation therapy setting, nine respondents (41%) reported that power outages “Rarely” contribute to treatment interruptions. Seven respondents (32%) mentioned that power outages “Sometimes” contribute to treatment interruptions. None of the respondents reported that power outages “Often” or “Always” contribute to treatment interruptions. Eleven respondents (50%) indicated that staffing “Rarely” contributes to treatment interruptions. Five respondents (23%) mentioned that staffing “Sometimes” contributes to treatment interruptions. Fourteen respondents (64%) reported that machine downtime “Sometimes” contributes to treatment interruptions. Eleven respondents (50%) mentioned that patient‐related issues “Sometimes” contribute to treatment interruptions. Eleven respondents (50%) indicated that natural disasters “Rarely” contribute to treatment interruptions, reflecting infrequent occurrences. None of the respondents reported that natural disasters “Often” or “Always” contribute to treatment interruptions. The frequency distribution of various factors that contribute to treatment interruptions in a radiation therapy setting is shown in Figure S1.

Table 1 presents the frequency of peer‐review chart rounds in a radiation therapy setting, as well as the frequency with which physician peer‐review file/chart rounds are conducted, seventeen respondents (77%) reported having peer‐review chart rounds, while five (23%) indicated the absence of such rounds. Respondents were asked to specify how often physician peer review file/chart rounds are conducted under two different scenarios: before the first fraction of treatment and within the first five fractions of treatment, 59% reported these always occur before the first fraction of treatment, and 5% reported they always occur within the first five fractions. However, 23% and 27% reported these rounds never occur in the respective scenarios. In terms of participation of various professionals in peer‐review chart round meetings in a radiation therapy setting radiation oncologists were the most frequent participants (91%), followed by medical physicists (50%), residents (36%), radiation therapists (32%), and oncology nurses (9%).

**TABLE 1:** The peer‐review chart rounds in the clinic.

Questions	Yes	No
Do they have peer‐review chart rounds?	17	5
How often are physician peer review file/chart rounds conducted?		
1. Before 1st fraction of treatment		
▪ Never	5	
▪ Rarely	1	
▪ Sometimes	1	
▪ Often	2	
▪ Always	13	
2. Within the first 5 fraction		
▪ Never	6	
▪ Rarely	2	
▪ Sometimes	9	
▪ Often	4	
▪ Always	1	
Who participates in physician peer‐review chart rounds meetings?		
▪ Radiation Oncologists	20	
▪ Medical Physicists	11	
▪ Oncology Nurses	2	
▪ Residents (Physician and Physicists)	8	
▪ Radiation Therapists	7	

*Note*: The “Yes” and “No” columns indicate the presence or absence of peer‐review chart rounds. It further elaborates on the frequency of these rounds within the first fraction of treatment or within the first 5 fractions. Additionally, it lists the healthcare professionals who participate in physician peer‐review chart round meetings.

Table 2 presents the use of systems for documenting, tracking, analyzing, and trending patient safety‐related incidents in radiation oncology clinics, as well as the reporting of incidents to the IAEA Safety in Radiation Oncology (SAFRON) program. Nineteen out of 22 respondents (86%) reported that their radiation oncology clinics use a system within the clinic. On the other hand, three respondents (14%) reported that their clinics do not use. Ten out of 22 respondents (46%) reported that their clinics utilize a hospital‐wide system for documenting, tracking, analyzing, and trending patient safety‐related incidents. On the other hand, twelve respondents (56%) indicated that their clinics do not utilize a hospital‐wide system. In the case of reporting incidents to IAEA SAFRON, seven out of 22 respondents (32%) reported that they do report patient safety. Fifteen respondents (68%) indicated that they do not report incidents to the IAEA SAFRON program.

**TABLE 2:** Use of systems for documenting, tracking, analyzing, and trending patient safety‐related incidents in radiation oncology clinics and reporting to IAEA SAFRON Program.

Questions	Yes	No
Does your clinic use a system of documenting, tracking, analyzing, and trending patient safety related incidents in radiation oncology?		
▪ System within the clinic (e.g., spreadsheet or paper)	19	3
▪ Hospital‐wide system	10	12
Do you report incidents to the IAEA Safety in Radiation Oncology (SAFRON)?	7	15

*Note*: The “Yes” and “No” columns reflect the responses to each respective question.

### 3.5 Disease types treated

Gynecologic cancer was rated “Always” by 82% of respondents, and breast cancer was rated “Always” by 68% of respondents, indicating these are the most frequently treated conditions in these clinics. In contrast, bone marrow transplants with total body irradiation were rated “Never” by 18 of the respondents, indicating they are rare occurrences. This suggests that breast cancer cases are a significant focus across facilities, reflecting its higher prevalence and clinical priority. Treatment frequency across various disease sites in clinics shown in Figure S2.

### 3.6 QA

The IAEA guidelines are the most used, with 19 out of 22 respondents (86%) reporting their use. AAPM TG 142 guidelines are frequently used, with 16 out of 22 respondents (73%) reporting their use. AAPM TG 40 guidelines are used by 11 out of 22 respondents (50%), and local guidelines for QA are used by 8 out of 22 respondents (36%). The frequency distribution of guidelines and recommendations used for QA in radiation therapy is shown in Figure S3.

Table 3 presents the results of routine QA checks conducted in a radiation therapy setting. For daily pre‐treatment QA checks, these assessments were reported by 20 respondents (91%), while 2 respondents (9%) did not report conducting them. The performance of these checks was attributed to 9 Radiation Therapists (41%), 6 Qualified Medical Physicists (27%), and 7 Medical Physics Residents/Trained Personnel (32%). Monthly QA checks were universally conducted by all 22 respondents (100%), with 13 (59%) being Qualified Medical Physicists, and 8 (36%) were Medical Physics Residents/Trained Personnel responsible for conducting the monthly QA checks. In annual QA checks, participation was observed among all 22 respondents (100%), with 17 Qualified Medical Physicists (77%) and 5 Medical Physics Residents/Trained Personnel (23%) responsible for performing the annual QA checks.

**TABLE 3:** The clinic's QA practices and procedures related to radiation therapy among 19 clinics.

Questions	Yes	No
Does the clinic perform daily QA testing before treatment?	20	2
If yes, who performs these tests?		
▪ Radiation Therapist		
▪ Qualified Medical Physicist (QMP)	6	
▪ Medical Physics Resident or Trained Personnel	7	
Does the clinic perform monthly QA for its delivery systems?	22	0
If yes, who performs these tests?		
▪ Medical Physics Resident or Trained Personnel	8	
▪ Qualified Medical Physics	13	
▪ Other: Both	1	
Does the clinic perform annual QA for its delivery systems?	22	0
If yes, who performs these tests?		
▪ Qualified Medical Physics	17	
▪ Medical Physics Resident or Trained Personnel	5	
▪ Other: Both	4	
Does the clinic have written QA procedures for the following?		2
▪ Pre‐ Treatment QA	11	
▪ Daily QA	19	
▪ Monthly QA	18	
▪ Annual QA	17	
How often are these output checks conducted?		
▪ Daily	8	
▪ Weekly	4	
▪ Bi‐weekly	2	
▪ Monthly	9	
▪ Annually	1	
Is beam calibration verified using the following methods?		
▪ Solid Water Phantom	12	
▪ None	4	
▪ Other: Water Phantom	6	
How often are the ionization chamber and electrometer calibrated externally?		
▪ Yearly	21	
▪ Over the year	1	
How often are the safety interlocks inspected?		
▪ Daily	19	
▪ Monthly	3	
▪ Annually	0	

*Note*: The “Yes” and ‘No’ columns indicate whether specific QA activities are performed, and the subsequent sections detail who conducts these tests and their frequencies, and how often these measurements are conducted.

Abbreviation: QA, quality assurance.

Respondents were also asked about the frequency of calibrating their ionization chamber and electrometer outside the facility. The majority, 21 out of 22 (96%), reported annual calibration outside the radiation therapy facility, while one respondent (5%) indicated calibration “over the year”. For safety interlocks, majority of respondents, 19 out of 22 (86%), reported conducting daily checks of their safety interlocks. Three respondents (14%) indicated that they perform monthly checks of their safety interlocks. None of the respondents reported performing annual checks of their safety interlocks.

In the case of verifying beam calibration in radiation therapy, the most common method is the use of a Solid Water Phantom, reported by 12 out of 22 respondents (55%). Water Phantoms are used by 6 out of 22 respondents (27%). Four respondents (18%) do not use any specific method for beam calibration verification.

In terms of utilization of various measuring devices and tools for constancy checks, the Ionization chamber and Electrometer are the most used, with utilization reported by 18 out of 22 respondents (82%). Solid Water Phantoms are employed by 15 out of 22 respondents (68%). An Array Detector is utilized by 9 out of 22 respondents (41%). Gafchromic Film is employed by 4 out of 22 respondents (18%). EPID Portal Imagers are used by 3 out of 22 respondents (14%). Water Phantoms are employed by 7 out of 22 respondents (32%). Traditional Film is the least commonly used, with utilization reported by only 1 out of 22 respondents (5%). The frequency distribution of various measuring devices and tools used for constancy checks is shown in Figure S4A.

For measuring devices and tools for dosimetric QA, Scanning Water Tanks are the most used measuring devices for dosimetric QA among the respondents, with 20 out of 22 respondents (91%) reporting their use. Calibrated Ionization Chambers are frequently utilized, with 18 out of 22 respondents (82%). Solid Water Phantoms are used by 9 out of 22 respondents (41%). 2D/3D Diode Arrays are employed by 7 out of 22 respondents (32%). Array Detectors are used by 5 out of 22 respondents (23%). The frequency distribution of various measuring devices and tools used for dosimetric QA is shown in Figure S4B.

For the utilization of mechanical checks on specific tests using various tools and indicators, Laser Localization is the most commonly employed mechanical check, with utilization reported by 21 out of 22 respondents (96%). The Optical Distance Indicator and Collimator Size Indicator tests were utilized by 19 out of 22 respondents (86%). Gantry and table checks were the least commonly performed, with utilization reported by only 2 out of 22 respondents (9%). The frequency distribution of mechanical checks on specific tests using various tools and indicators is shown in Figure S4C.

For QA tests for imaging, Planar MV (Electronic Portal Imaging Device) Imaging is the most performed QA test, with 16 out of 22 respondents (73%). Films are used for imaging QA tests by two respondents (9%). Planar kV and MV (EPID) Imaging QA tests are performed by only one respondent (5%). None of the respondents reported performing QA tests specifically for Cone Beam CT (CBCT) imaging. Three respondents (14%) reported performing no specific QA tests for imaging. The frequency distribution of the QA tests for imaging is shown in Figure S4D.

For methods used for performing secondary MU calculation checks. Commercial MU Calculation Software is used “Always” by 50% of the respondents. The majority (46%) of respondents use custom and hand calculation software “Rarely.” The frequency distribution of various methods used for performing secondary MU calculation checks is shown in Figure S5.

Table 4 presents the patient‐specific QA for Intensity‐Modulated Radiation Therapy (IMRT) or Volumetric Modulated Arc Therapy (VMAT). Respondents were asked about their patient‐specific QA (PSQA) practices, with options “Yes,” “No,” or “Not Sure.” Of the respondents, 18 out of 22 (82%) reported performing PSQA, while one (5%) indicated they did not, and three (14%) were unsure. For those who perform PSQA (as mentioned earlier), pre‐treatment QA measurements were conducted. The majority (41%) reported performing these measurements “Always,” followed by “Often” (14%), “Sometimes” (9%), and “Never” (36%). None reported performing them “Rarely.” Regarding the method used for PSQA in IMRT/VMAT, Portal EPID dosimetry was the most common, used by 12 out of 22 (55%) respondents. 2D array devices for field‐by‐field verification were used by 7 out of 22 (32%), followed by Ion chambers for point dose verification (23%), and 3D array phantoms (23%). Slab phantoms were the least commonly used (5%), and Film dosimetry was used by one respondent (5%) for PSQA.

**TABLE 4:** The availability and patient‐specific QA practices related to IMRT or VMAT treatment at the 19 clinics.

Questions	Yes	No	Not sure
Is IMRT or VMAT treatment available at the clinic?	22	0	
If “Yes”, is PSQA testing conducted for IMRT or VMAT?	18	1	3
If the response is “No”, what's preventing VMAT or IMRT implementation and treatment planning in the institution?		No IMRT Phantom	
What method is employed for PSQA in IMRT/VMAT?			
▪ Ion chamber for point dose verification	5		
▪ 2D array device for field‐by‐field verification	7		
▪ 3D array phantoms	5		
▪ Portal EPID dosimetry	12		
▪ Film	1		
▪ Other: Slab Phantom	1		
If “Yes”, how often are pre‐treatment QA measurements done before or after the first fraction?			
▪ Always	9		
▪ Often	3		
▪ Sometimes	2		
▪ Rarely	0		
▪ Never	8		

*Note*: The “Yes,” “No,” and “Not Sure” columns reflect the responses to each respective question.

Abbreviations: PSQA, patient‐specific QA; QA, quality assurance; IMRT, intensity‐modulated radiation therapy; VMAT, volumetric modulated arc therapy.

### 3.7 Equipment/Tool availability

#### 3.7.1 Dosimetry and measurement devices

Solid Water Phantoms and Scanning Water Tanks are the most used devices, utilized by 19 out of 22 respondents (86%). The EPID Portal Imager, Front Pointer Sets, and Graph Paper are each utilized by 18 out of 22 respondents (82%). Film is utilized by 17 out of 22 respondents (77%), while both Array Detectors and Ionization Chambers are used by 16 out of 22 respondents (73%). 2D/3D Diode Arrays were reported by 13 out of 22 respondents (59%). TBI/TSET Phantom is the least frequently used device, with only 1 out of 22 respondents (5%). For film dosimetry usage, Gafchromic films are the most commonly used film type, with 19 out of 22 respondents (86%), while Radiographic films are reported by 12 out of 22 respondents (55%). The frequency distribution of dosimetry and measurement devices in the clinics is shown in Figure S6A.

#### 3.7.2 Image guidance

Onboard EPID‐based MV imaging is the most commonly used, with 19 out of 22 respondents (86%) reporting its use. On‐board 2D kV imaging is reported by 6 out of 22 respondents (27%). Onboard 3D CBCT (kV or MV) imaging is employed by 4 out of 22 respondents (18%). Film‐based MV imaging is used by 2 out of 22 respondents (9%). The utilization of CT on rails and surface‐guided imaging was not reported by any of the respondents. The utilization of various image‐guidance techniques is shown in Figure S6B.

#### 3.7.3 Motion management

In‐room video camera of the patient viewed at the treatment console is the most commonly used, with their utilization reported by 16 out of 22 respondents (73%). Fiducials are employed by 9 out of 22 respondents (41%). The absence of any specific technique for monitoring or controlling patient positioning during radiation therapy is noted among 5 out of 22 respondents (23%). DIBH is utilized by 1 out of 22 respondents (5%), while Active Breathing Control (ABC) is not reported as being used by any of the respondents. The utilization of techniques for monitoring and controlling patient positioning during radiation therapy is shown in Figure S6C.

#### 3.7.4 Immobilization device/technique

Headrests are the most commonly used, with all 22 respondents (100%) indicating their utilization. Body conformal immobilization devices are used by 20 out of 22 respondents (91%), and thermoplastic masks are reported by 21 out of 22 respondents (96%). Setup for total body irradiation is the least commonly used device, with only 2 out of 22 respondents (9%). The utilization of patient immobilization and positioning devices is shown in Figure S6D.

## 4 DISCUSSION

The survey provides insights into various aspects of medical physics practice, including training and employment, the availability of treatment modalities and machines, QA practices, equipment availability, and quality management and safety practices. From the 22 respondents working in various clinics and hospitals around the Philippines, the majority had received 1–3 years of training, followed by 4–7 years and 8–15 years of training. Additionally, 14% were currently in the training phase, demonstrating the continuous demand for medical physicists in the field. Only one participant had practiced for more than 15 years. In terms of employment institutions, private hospitals employed the majority of medical physicists, while government hospitals accounted for 46% of the cohort.

The Philippines faces a significant shortage of radiation oncology medical physicists (ROMPs), with only 103 working in the country, averaging just 2 MPs per facility, below the recommended 3 to 4,^29^ and fewer than 20 holding certifications in radiation oncology medical physics (CMP‐ROMP), necessitating collaborative strategies to address the pressing workforce shortage and meet the growing demand for radiation therapy services.^7^ These findings highlight the dynamic nature of the medical physics workforce, characterized by a mix of experience levels and employment settings, which may have implications for workforce planning and training programs in the country. The data highlight that qualified medical physicists (CMP‐ROMP) predominantly perform initial plan checks (68% always or often) and weekly physics checks (77% always or often). In rare cases (5% for initial checks, 9% for weekly checks), medical physics trainees take on these roles, primarily in provincial clinics in the Philippines where CMP‐ROMP act as consultants due to a shortage of certified professionals. While CMP‐ROMP focuses on consulting or advanced oversight, MPTs handle operational tasks, ensuring that plan quality and deliverability are maintained. The findings reveal differences in QA practices between CMP‐ROMPs and MPTs. While the majority (55%) of CMP‐ROMPs perform QA checks “Always,” reflecting their training and expertise, a significant proportion (36%) of MPTs reported performing these checks “Never,” raising concerns about their readiness. These results emphasize the need for enhanced training programs for MPTs, focusing on QA responsibilities, alongside mentorship opportunities with CMP‐ROMPs to build their skills and confidence. Seventeen respondents (77%) reported having peer‐review chart rounds, indicating a commitment to QA. However, while only 5% maintain this practice within the first five fractions, the variability in the frequency of rounds before and within the first five fractions of treatment indicates potential areas of improvement in review protocols.

The survey results show that the IAEA guidelines (86%), AAPM TG 142 (73%), and AAPM TG 40 (50%) are the most commonly used frameworks for radiotherapy QA. However, local guidelines, such as the Philippine Department of Health's Administrative Order No. 2013–0031,^30^ play a critical role, with 36% of respondents reporting their use. This order establishes comprehensive regulations for therapeutic x‐ray facilities using medical LINACS to ensure high‐quality radiotherapy. It emphasizes minimizing radiation risks to patients, healthcare workers, and the public through strict adherence to safety standards, compliance with the FDA Act of 2009, and rigorous quality management practices, enhancing treatment efficacy while safeguarding health and safety.

The study assessed the availability of treatment modalities in 19 clinics. All clinics utilized LINACs. Only one clinic employed a Co‐60 Unit with a last source exchange in 2015. Regarding the use of brachytherapy, High Dose Rate (HDR) was the predominant modality, utilized by 16 clinics (73%), while LDR was reported by only one clinic (5%). None of the clinics reported the use of the “Permanent” radiation therapy modality. These findings highlight the common use of the LINAC machine and the preference for HDR brachytherapy among the surveyed clinics, which may have implications for equipment procurement and resource allocation in the country. As of early 2023, the Philippines has made significant progress in expanding its radiotherapy facilities, with a total of 51 centers in operation.^7^ The count of radiotherapy facilities has increased by more than two‐fold since 2015, when there were only 22 such facilities available.^4^ However, there remains a notable disparity in the distribution of these facilities across the country. The majority of these radiotherapy facilities (39%) are concentrated in the NCR (Metropolitan) region, while 33% are located in various parts of Luzon, and 14% are situated in both Visayas and Mindanao. This concentration of facilities in highly urbanized areas, particularly in NCR and Luzon, creates challenges for patients residing in other regions who may need to travel long distances to access essential cancer treatment services. The distribution of radiotherapy practices among the 19 clinics surveyed in the Philippines reveals significant regional disparities, with NCR dominating access to advanced modalities. IMRT and 3D‐CRT are the most widely available among clinics, while advanced techniques like SBRT and SRS, and specialized treatments such as VMAT, TBI, and IORT are commonly practiced in NCR, leaving other regions underserved and highlighting the absence of Proton Therapy and TSE nationwide. NCR leads in the use of advanced technologies, reflecting better infrastructure and accessibility. In contrast, some parts of Luzon, Visayas, and Mindanao show limited usage, with most activity concentrated in IMRT and 3D‐CRT practices. Overall, the data highlights significant disparities in access to advanced radiation therapy techniques, with underserved regions still reliant on legacy technologies. This limits the availability of cancer treatments for patients outside the capital region.

The 2020 Globocan data emphasizes the critical importance of addressing the cancer burden in the Philippines. With an estimated 153,751 new cancer cases per year, each radiotherapy facility is tasked with treating approximately 2901 patients annually. Among the 19 clinics surveyed, there is a wide range in the number of patients treated, with a minimum of 120 patients and a maximum of 15 000 patients a year. It's worth noting that one public hospital stands out as having a Co‐60 teletherapy machine and treating 250 patients per year. This information highlights both the demand for radiotherapy services and the capacity constraints faced by many facilities. To address these disparities and ensure that all Filipinos have equitable access to radiotherapy services, it is essential for decision‐makers to adopt a comprehensive framework. This framework should prioritize the equitable distribution of advanced radiotherapy technologies and techniques throughout the country, considering the unique needs of both urban and rural areas. Additionally, the development of a strategic national plan, informed by careful assessment of national and regional needs, is crucial for efficiently allocating resources and expanding radiotherapy infrastructure where it is most needed.

In terms of the availability of imaging modalities in clinics, CT emerged as the most common imaging modality, with a 100% utilization rate across all surveyed clinics. MRI and PET/CT were employed by 59% and 23% of clinics, respectively. SPECT was used by 14% of clinics. These findings provide an insight on the critical role of CT in treatment planning, underlining its common use as a diagnostic tool in the clinics surveyed. However, the varying adoption rates of advanced imaging modalities, such as MRI and PET/CT, indicate disparities in their integration into clinical practice. Furthermore, among the 19 clinics surveyed, it's noteworthy that 8 clinics had their CT simulator located in a separate clinic or within the Radiology Department. These insights underscore the importance of optimizing the accessibility of CT simulator within clinics to enhance the time efficiency during treatments.

In terms of the frequency of treating different disease sites, gynecologic and breast conditions were frequently treated, with breast cancer patients being treated “Always” or “Often”. Lung cancer treatment varied, with the highest percentage indicating that it is treated “Sometimes.” The study also highlighted that bone marrow transplants with total body irradiation were rare occurrences. These results align with the findings that breast cancer is the leading cancer case in the country for both sexes, according to Globocon,^1^ making it one of the most frequently treated disease sites. Furthermore, lung cancer is also prevalent in the country and is typically treated on “Sometimes”. Surprisingly, according to 2020 Globocon data,^1^ cervix uteri and ovary ranked as the 2nd and 5th most common new cancer cases in females in the country, emerging as the most frequently treated disease site from the survey. These findings offer valuable information for resource allocation and treatment planning within clinics.

For patient immobilization and positioning devices, there were high utilization rates for headrests, thermoplastic masks, and body conformal immobilization devices. In contrast, Setup for total body irradiation had lower utilization. The lower utilization rates of fiducials and DIBH suggest that these methods may not be as widely adopted in the surveyed clinics. The utilization of Setup for total body irradiation suggests that this specialized technique is less common in the surveyed clinics, which aligns with the rarity of total body irradiation cases mentioned in the initial results. In the Philippines, there's no local production of immobilization devices like masks, headrests, and cushions. This forces some hospitals to import or improvise, which can be costly and challenging due to the country's location. Healthcare professionals’ resort to creative solutions, making clones of headrests from foam or wood and sterilizing and remolding masks for reuse, especially for indigent patients. For instance, they use jump ropes from local toy stores for shoulder depression during head and neck treatments. Provide alternatives to costly immobilization foam, where radiation oncologists devised a cost‐effective which can be molded around patients for alpha‐cradles.^4^

A significant majority of the participants, specifically 82%, reported that they perform PSQA, emphasizing the importance of this practice in radiation therapy. Among those who conducted pre‐treatment QA measurements, 41% did so “Always,” indicating a commitment to rigorous QA protocols. Portal EPID dosimetry emerged as the most frequently employed method for PSQA in IMRT/VMAT, utilized by 55% of the respondents, highlighting its prevalence in clinical practice. Slab phantoms were the least commonly used, and Film dosimetry had limited representation. These findings underscore the significance of PSQA in modern radiation therapy.

The study provided extensive insights into QA practices, including routine checks, beam calibration, dosimetric QA, mechanical checks, imaging QA, and secondary MU calculation checks. These comprehensive findings highlight the QA procedures implemented in radiation therapy clinics, ensuring the safety and precision of treatments. Also, the study explored factors contributing to treatment interruptions, such as power outages, staffing, machine downtime, patient‐related issues, and natural disasters. Power outages and natural disasters were reported to have limited impact, while staffing and machine downtime were occasionally cited as contributors. Majority of clinics (86%) employ internal systems for incident documentation and tracking, underscoring their commitment to patient safety. However, only a minority (32%) engage in external reporting to the IAEA SAFRON program, indicating potential room for improvement in sharing safety‐related data at the international level and promoting broader safety standards within the field of radiation oncology.^31^ These results emphasize the importance of proactive measures to minimize treatment interruptions.

The study investigated the availability of dedicated research time, research resources, collaboration willingness, and receptiveness to research collaboration. Findings indicated limited availability of dedicated research time but a strong willingness to collaborate with colleagues from other institutions and abroad. This highlights the potential for collaborative research efforts to advance the field, despite resource constraints.

## 5 CONCLUSION

The survey results provide a comprehensive overview of various aspects of medical physics practice, including training, equipment availability, treatment modalities, QA, and safety practices. The data collected can be valuable for understanding the current landscape of radiation therapy and guiding future improvements and resource allocation within clinics and institutions. The study reveals the dynamic nature of the medical physics workforce, varying experience levels, and employment settings, calling for adaptable workforce planning and training programs. Disparities in treatment facility distribution result in many cancer patients, particularly those outside major cities, lacking essential care access. While the growth in the number of radiotherapy facilities in the Philippines is a positive development, addressing the disparities in distribution and ensuring equitable access to cancer treatment services remain critical objectives. Achieving this goal requires a multi‐faceted approach, including strategic planning, resource allocation, and a commitment to providing advanced radiotherapy options to all Filipinos, regardless of their geographic location. The importance of CT in treatment planning is emphasized, though uneven adoption rates of advanced imaging modalities present challenges. Breast and gynecologic conditions are commonly treated, with lung cancer's high prevalence in the country. This data offers valuable information for resource allocation and treatment planning within clinics. The study underscores the significance of rigorous QA practices and the resource challenges in device/equipment availability. Future directions include addressing disparities to ensure equitable cancer treatment access, optimizing the utilization of advanced imaging modalities, enhancing QA practices through ongoing training, promoting research collaboration, and optimizing resource utilization within clinics.

## AUTHOR CONTRIBUTIONS

*Manuscript writing, data collection and processing, analysis and interpretation, and literature search*: John Paul C. Cabahug. *Critical review and interpretation*: Ramon Carlo Cruzpero. *Concept, design, experimental material, and resources*: Luis E. Fong de los Santos. *Concept, design, experimental material, and resources*: Eric C. Ford. *Supervision and leadership, critical review, concept, design, experimental material, analysis and interpretation, and resources*: Afua A. Yorke.

## CONFLICT OF INTEREST STATEMENT

The authors declare no conflicts of interest.

## Supporting information



Supporting Information

Supporting Data Online Survey Form
