# Supplementary material for: Radiotherapy medical physics in the Philippines: A contemporary overview
Source: J Appl Clin Med Phys. 2025 May 31;26(7):e70129. doi: 10.1002/acm2.70129 (PMC12256689; doi:10.1002/acm2.70129)
Supplement: Supplementary file 1 — Supporting Information [file ACM2-26-e70129-s002.docx]

**
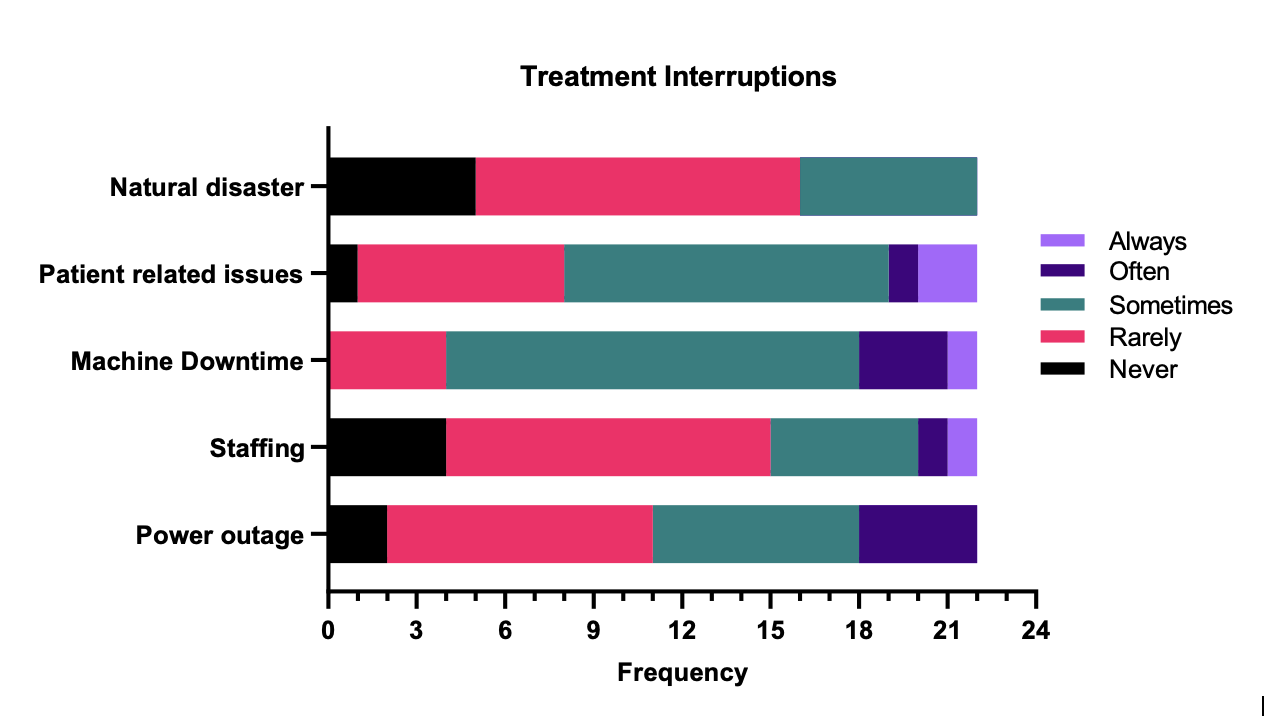
**

**Supplementary Figure 1.** Frequency distribution on various factors that contribute to treatment interruptions in a radiation therapy setting using a 5-point Likert scale (1 = Never, 2 = Rarely, 3 = Sometimes, 4 = Often, 5 = Always).

**Supplementary Figure 2.** Frequency distribution across various disease sites treated across clinics using a 5-point Likert scale (Never =1, Rarely =2, Sometimes =3, Often =4, Always =5).

**Supplementary Figure 3.** Frequency distribution of various Quality Assurance (QA) protocols/guidelines in radiation therapy practices among 19 clinics.

**Supplementary Figure 4A-D.** Frequency distribution of using various Quality Assurance (QA) tools for constancy checks (A), dosimetry (B), mechanical checks (C), and imaging QA tests (D) in radiation therapy practices among 19 clinics.

**Supplementary Figure 5.** Frequency distribution of secondary Monitor Unit (MU) calculation check methods using a 5-point Likert scale (1 = Never, 2 = Rarely, 3 = Sometimes, 4 = Often, 5 = Always).

**Supplementary Figures 6A-D**. Frequency distribution of equipment/tool availability for quality assurance devices (A), image guidance techniques (B), motion management strategies (C), and immobilization devices (D) in radiation therapy practices among 19 clinics.

**Supplementary Table 1.** The availability of dedicated research time, research resources, and the willingness to collaborate in an institution. The 'Yes' and 'No' columns reflect the responses to each respective question.

| **Questions** | **Yes** | **No** |
| --- | --- | --- |
| Does the institution provide dedicated research time? | 2 | 20 |
| If yes, do they have research resources like funding or international collaborations? | 1 | 21 |
| If no, are they open to collaborating with colleagues from other institutions and abroad? | 22 | 0 |
| Are they receptive to being contacted for research collaboration? | 22 | 0 |

**Supplementary Table 2.** The qualitative assessment of equipment needs and priorities expressed by the respondents in their respective clinics.

| **Equipment** | **Qualitative Assessment** |
| --- | --- |
| 1. New Software and New PCs | Respondents expressed a need for new software and computers, which could enhance the efficiency of treatment planning and data management. |
| 1. 6-Degree of Freedom Couch | The request for a 6-degree of freedom couch suggests a desire for advanced treatment positioning capabilities, which can be particularly beneficial for precise patient positioning during radiation therapy. |
| 1. Deep Inspiration Breath Hold (DIBH) | Respondents highlighted the importance of DIBH technology in managing cases where the target moves due to involuntary body motions, such as breathing. This technology can significantly improve the accuracy and safety of treatments, especially in Breast and Lung RT. |
| 1. CT Simulator | Some clinics have their CT simulator located in either other clinics or the radiology department. This can contribute to more efficient workflows and treatment planning. |
| 1. EPID-Specific QA Device | The request for an EPID-specific QA device indicates a focus on improving quality assurance. |
| 1. Additional Big Bore CT scan | Some respondents expressed the need for an additional Big Bore CT scan, particularly for accommodating cancer patients with larger body frames. This reflects a commitment to addressing the diverse needs of patients. |
| 1. Specific Dosimetry QA Devices | The desire to include specific dosimetry QA devices in the purchase of new LINACs reflects a commitment to research and quality assurance in radiation therapy. |
| 1. Software for Dose Calculation | This highlights the importance of accurate treatment planning and dose calculation in radiation therapy. |
| 1. Ready Contour Software | The mention of ready contour software suggests a need for tools that streamline the contouring process during treatment planning, which can improve accuracy and efficiency. |
