# Supplementary material for: Radiotherapy medical physics in the Philippines: A contemporary overview
Source: J Appl Clin Med Phys. 2025 May 31;26(7):e70129. doi: 10.1002/acm2.70129 (PMC12256689; doi:10.1002/acm2.70129)
Supplement: Supplementary file 2 — Supporting Data Online Survey Form [file ACM2-26-e70129-s001.docx]

**I. Online Survey Form via REDCap**

**IV. Institutional Review Board (IRB) Exemption**

**II. Invitation Letter**
